# Hepatocyte Growth Factor Effects on Mesenchymal Stem Cells Derived from Human Arteries: A Novel Strategy to Accelerate Vascular Ulcer Wound Healing

**DOI:** 10.1155/2016/3232859

**Published:** 2015-12-14

**Authors:** Sabrina Valente, Carmen Ciavarella, Emanuela Pasanisi, Francesca Ricci, Andrea Stella, Gianandrea Pasquinelli

**Affiliations:** ^1^Department of Experimental, Diagnostic and Specialty Medicine (DIMES), University of Bologna, Via Massarenti 9, 40138 Bologna, Italy; ^2^Cardiovascular Tissue Bank-Immunohematology and Transfusion Medicine, University Hospital St. Orsola-Malpighi, Polyclinic of Bologna, Via Massarenti 9, 40138 Bologna, Italy

## Abstract

Vascular ulcers are a serious complication of peripheral vascular disease, especially in diabetics. Several approaches to treat the wounds are proposed but they show poor outcomes and require long healing times. Hepatocyte Growth Factor/Scatter Factor (HGF/SF) is a pleiotropic cytokine exerting many biological activities through the c-Met receptor. This study was aimed at verifying whether HGF/SF influences proliferation, migration, and angiogenesis on mesenchymal stem cells isolated from human arteries (hVW-MSCs). hVW-MSCs were exposed to NIBSC HGF/SF (2.5, 5, 10, and 70 ng/mL) from 6 hrs to 7 days. HGF and c-MET mRNA and protein expression, cell proliferation (Alamar Blue and Ki–67 assay), migration (scratch and transwell assays), and angiogenesis (Matrigel) were investigated. hVW-MSCs displayed stemness features and expressed HGF and c-MET. HGF/SF did not increase hVW-MSC proliferation, whereas it enhanced the cell migration, the formation of capillary-like structures, and the expression of angiogenic markers (vWF, CD31, and KDR). The HGF/SF effects on hVW-MSC migration and angiogenic potential are of great interest to accelerate wound healing process. Local delivery of HGF/SF could therefore improve the healing of unresponsive vascular ulcers.

## 1. Introduction

The lower limb ulceration has prevalence of about 3% in the adult population over 65 years [1]. Foot ulcers mainly affect the arterial system and are particularly severe and devastating among diabetic patients, where they tend to become chronic. For this reason, foot ulcers significantly affect the expectancy and quality life of such patients, as well as healthcare expenditures. Arterial ulcers are a consequence of diffuse atherosclerotic narrowing of the leg arteries resulting in a significant reduction of blood flow to the lower limb. The main therapy consists of restoring blood flow by angioplasty or bypass and the removal of the debridement and the necrotic tissue to accelerate the wound healing. Additional treatments have been recently introduced, such as biological dressing, physical therapy (hyperbaric oxygen and negative pressure therapy), and compression therapy [2]; however, clinical results are still less than optimal.

To date, surgical revascularization remains the milestone of any arterial ulcer treatment; however, blood flow restoration should be associated with treatments aimed at reactivating ineffective autologous healing processes, such as inflammatory cell responses and local angiogenesis whose ineffectiveness is responsible for incomplete and delayed wound healing. In this respect, the use of gene and cell therapies [3] and natural or synthetic engineered matrices [2, 4] has gained interest in the scientific community; as an example, interesting results on the complete wound healing were seen after transplantation of biomimetic tissue engineered dermis substitutes with allogeneic keratinocytes and fibroblasts cells inside ulcerated tissue [5, 6].

Interestingly, both animal and human studies advocate the adult mesenchymal stem cells derived from bone marrow [7, 8] as well as adipose tissue [9–11] as ideal candidates to treat nonhealing wound due to their ability to differentiate in multiple mesengenic lineages and their capability to facilitate angiogenesis through the secretion of proangiogenic growth factors, even if their exact contribution to wound healing has not been completely understood.

Human derived autologous platelet-rich plasma (PRP) is also a promising wound healing treatment, due to the mitogenic and chemoattracting effects exerted by growth factors released at high concentrations after platelet *α*-granules degranulation [12]. Some case reports and few clinical trials [13–15] showed positive effects of PRP on reepithelialization of nonhealing wounds.

Alternatively, single or combined growth factors including Platelet-Derived Growth Factor (PDGF), Vascular Endothelial Growth Factor (VEGF), Transforming Growth Factor-beta (TGB-beta), Fibroblast Growth Factor (FGF), Epidermal Growth Factor (EGF), and Granulocyte Macrophage-Colony Stimulating Factor (GM-CSF) are largely studied [16, 17], thanks to their multiple effects on wound healing including cell proliferation and mobilization, extracellular matrix production, and angiogenesis even though different outcomes are reported. In addition, the Hepatocyte Growth Factor/Scatter Factor (HGF/SF) is a multifunctional cytokine involved in numerous biological responses including cell proliferation/survival, angiogenesis, morphogenesis, and motogenesis [18] as well as inflammation and fibrosis inhibition [19]; these actions are exerted through its tyrosine kinase receptor, c-Met [20], that is primarily expressed in epithelial cells; however, few reports indicate that adult mesenchymal stem cells also express c-Met [21–23].

In this study, we tested the* in vitro* effects of HGF/SF on a multipotent mesenchymal stem cells population that our research group isolated from the vascular wall of adult human arteries (hVW-MSCs) [24]. The hVW-MSC exposure to HGF/SF could represent a strategy to improve and accelerate the wound healing process in foot ulcers. In particular, we investigated the HGF/SF effect on cell proliferation and viability using Alamar Blue assay and Ki-67 immunofluorescence staining, cell migration and motility abilities through scratch and transwell assays, and angiogenic potential to form capillary-like structures in a Matrigel assay.

## 2. Materials and Methods

### 2.1. Human Vascular Wall-Mesenchymal Stem Cell Isolation and Culture Condition

Vascular wall-mesenchymal stem cells (hVW-MSCs) isolated from human arteries were used according to the ethic protocol (APP-13-01) approved by the Local Ethics Committee of University Hospital St. Orsola-Malpighi of Bologna in Italy and with consent informed. Their isolation, characterization, and stemness investigation were performed using methods described elsewhere [24]. Briefly, the human arteries were enzymatically digested overnight at 37°C in a rotor apparatus using serum-free DMEM culture medium supplemented with 0.3 mg/mL Liberase type II (Roche, Milan, Italy) followed by filtration through 40–70–100 *μ*m nylon mesh cell strainer (Becton Dickinson; Franklin Lakes, NJ) seeded on collagen-coated flasks and cultured in complete DMEM plus 20% Fetal Bovine Serum (FBS) at 37°C in a humidified atmosphere of 5% CO_2_ for 3 days. After floating cells removal, adherent hVW-MSCs were expanded until confluence replacing the cell culture with fresh medium every 2-3 days. Immunophenotype, stemness features, and multilineage potential were investigated confirming their mesenchymal identity [24]. In this study, experiments were performed using cells taken at passages 3 and 4 and cultured in DMEM plus 10% of FBS with or without Hepatocyte Growth Factor/Scatter Factor (HGF/SF) (WHO Reference Reagent, HGF/SF, NIBSC code: 96/564, National Institute for Biological Standards and Control, Potter Bar, Hertfordshire, ENG 3QG) at different time (from 6 hrs to 7 days) and concentrations (from 2.5 to 70 ng/mL). Cell starvation with low percentage of serum (0.5% FBS) for 12 hrs was performed to induce cell cycle synchronization before pretreatment with 0.2 *μ*M PHA-665752 inhibitor (Tocris Bioscience, Bristol, UK) for 12 hrs, followed by HGF/SF addition. All experiments were executed in triplicate.

### 2.2. RNA Extraction and RT-PCR

RT-PCR was performed on hVW-MSCs to detect the basal expression of HGF and its receptor c-Met. Total RNA was extracted from hVW-MSCs using TRIreagent according to the manufacturer's instructions (TRIzol reagent; Invitrogen). Reverse transcription of 1 *μ*g of total RNA was carried out in a 20 *μ*L volume of reaction using a High Capacity Reverse Transcription Kit (Applied Biosystems, Carlsbad, CA, USA). Polymerase Chain Reaction (PCR) products were analyzed by electrophoresis on a 2% agarose gel, stained with ethidium bromide and photographed under ultraviolet light. All PCR product sizes were identified loading a 100-base-pair (bp) DNA ladder and normalized to GAPDH, used as housekeeping gene. The PCR primers were purchased from Sigma-Aldrich. Genes and respective primers are presented in Table 1.

### 2.3. Western Blot Analysis

Total cellular proteins were extracted by untreated and treated hVW-MSCs using lysis buffer (KH2PO4 0.1 M pH 7.5, NP-40 1%, and 0.1 mM *α*-glycerolphosphate, added with complete protease inhibitors cocktail, Roche Diagnostics) and quantified spectrophometrically with the Bio-Rad Protein Assay (Bio-Rad Laboratories, Hempstead, UK). Thirty *μ*g proteins were subjected to 8% SDS-PAGE and transferred to nitrocellulose membrane (GE Healthcare Life Sciences, Amersham) at 30 mA for 2 hrs and 30 minutes. The membrane was blocked with 5% nonfat dry milk in TBS-tween for 1 hour at room temperature (RT), incubated with primary antibodies against c-Met (1 : 500, Santa Cruz Biotechnologies), HGF (1 : 500, Santa Cruz Biotechnologies), and anti-*α*-actin (clone AC-74, Sigma-Aldrich) at 4°C overnight. Secondary antibodies (human anti-rabbit/mouse horseradish peroxidase-conjugated (GE Healthcare, Milan, Italy)) were used at 1 : 10000 dilutions for 1 h at room temperature (RT). Protein signal was detected using Westar *η*C chemiluminescent substrate (Cyanagen).

### 2.4. Cell Viability and Proliferation Evaluation

Cell viability was investigated using Alamar Blue fluorescence assay (Invitrogen, Milan, Italy). The hVW-MSCs were seeded in a 12-multiwell plate at the density of 3 × 10^4^ and cultured in complete DMEM with or without HGF/SF (2.5, 5, and 10 ng/mL) for 1, 3, and 7 days. Alamar Blue solution (10% v/v in cultured medium) was added to each well at the end of treatments and incubated for 4 hrs at 37°C according to the manufacturer's instruction. Alamar Blue fluorescence (Ex/Em = 540/590 nm) of three replicates in each well was measured in a Wallac VICTOR2 multiplate reader (Perkin Elmer, Milan, Italy). In addition, a standard polystyrene well was used to measure the background fluorescence; this fluorescence was subtracted from the reading of each well.

Cell proliferation was assessed using single immunofluorescence staining for cycling cells expressing Ki-67 protein. hVW-MSCs were plated at a density of 6 × 10^5^ on coverslip in 6-well plates in DMEM overnight to allow the cell confluence and treated with or without HGF/SF (2.5, 5, 10, and 70 ng/mL) for 6 and 24 hrs. In parallel experiments, additional cell-seeded glass was starved in 0.5% FBS for 12 hrs, pretreated with 0.2 *μ*M PHA-665752 inhibitor, and subjected to HGF/SF exposure at the same time and concentrations. Untreated cells were used as a control.

### 2.5. Immunofluorescence Staining

At the end of treatments, cells were washed, fixed, and permeabilized in 2% paraformaldehyde in PBS with 1% Tryton X-100 for 4 minutes at RT, blocked with 1% bovine serum albumin (BSA) for 30 minutes at RT to reduce nonspecific staining, and labeled with monoclonal antibody against nuclear transcription factor Ki-67 (1 : 100, Novocastra, Leica Microsystems, Wetzlar, Germany) and intermediate filament Vimentin (1 : 100, Dako Cytomation, Glostrup, Denmark). Samples were washed, stained with AlexaFluor-488 (1 : 250, Life Technology, Carlsbad, CA, USA) secondary antibody in the dark, and counterstained with Pro Long antifade reagent with DAPI (Molecular Probes, Milan, Italy). All incubations were performed for 1 hr at 37°C in a wet chamber; both antibodies were diluted in 1% BSA in PBS. Samples were observed and photographed in a Leica DMI6000 B inverted fluorescence microscope (Leica Micro-systems, Wetzlar, Germany). Negative control was performed by omitting the primary antibody and no fluorescence was detected. For each experimental condition, the number of Ki-67 intensely stained cells as well as DAPI stained nuclei was manually counted on ten random fields and their values were expressed in percentage as ratio of Ki-67 stained cells on total cells number.

### 2.6. *In Vitro* Wound Healing Assay

Cell migration was investigated using a scratch assay. HVW-MSCs were seeded in 12-multiwell plate at a density of 1 × 10^5^/well and grown until to confluence. Cell monolayers were wounded with a p200 pipette tip, washed with PBS to remove cell debris, and treated with complete DMEM containing HGF/SF (2.5, 5, 10, and 70 ng/mL) for 24 hrs. In further experiments, cells were starved, inhibited with 0.2 *μ*M PHA-665752, and cultured in presence of HGF/SF at the same time and concentrations. At the end of HGF/SF treatment for both experiments, cells were fixed in absolute methanol for 10 minutes, washed in PBS, stained with 0.1% Crystal Violet in 25% methanol for 30 minutes, and air-dried; all steps were performed at RT. The wound closure was observed under a phase-contrast light microscope (LM) equipped with a digital camera (Nikon), acquiring images for each sample at time 0 and 24 hrs, respectively, using Software NIS-elements D3.2 Nikon (Tokyo, Japan). Computer-assisted image analysis (Image-Pro Plus software, Media Cybernetics, http://www.mediacy.com/) was employed to perform the quantification of the area of cells migrated into scratched area as well as the total wounded area in three different fields. Values were expressed in percentage as ratio of migrated cell area on total scratched area. Additional cell-seeded glasses were used for Vimentin intermediate filaments immunofluorescence staining.

### 2.7. Transwell Migration Assay

The cellular ability to migrate through a porous membrane under the influence of a chemoattractant factor was evaluated using transwell chambers (Costar, Corning Incorporated, NY, USA). Briefly, 500 *μ*L of DMEM with 10% FBS with or without chemoattractant HGF/SF (10 ng/mL) was placed below the polycarbonate membrane with 8 *μ*m pores while 300 *μ*L of cellular suspension containing 2.5 × 10^4^ hVW-MSCs in growth medium was plated on the upper layer of the membrane for 24 hrs at 37°C in incubator. After the manual removal of the nonmigrated cells from the upper side of the membranes, the inserts were detached from the plastic support using a scalpel, fixed, stained with 0.1% Crystal Violet for 30 minutes at RT, and mounted on glass slide. The cells migrated in the lower layer were observed using a Leitz Diaplan LM (Wetzlar, Germany) equipped with a video camera (JVC 3CCD video camera, KY-F55B, Jokohama, Japan). Digital images were acquired at 10x of magnification using Image-Pro Plus 6 software (Media Cybernetics). Additional membranes were processed for SEM analysis.

### 2.8. Scanning Electron Microscopy (SEM)

For SEM, samples were rinsed in 0.15 M phosphate buffer to remove the culture medium, fixed in 2.5% buffered glutaraldehyde (TAAB Laboratories, UK) overnight at 4°C, washed again in phosphate buffer, postfixed in 1% osmium tetroxide in 0.1 M phosphate buffer, wet in distilled water, and dehydrated with increasing ethanol concentrations (70–100%). Each step was performed at RT for 15 minutes. For drying the samples, they were immersed before in a solution of 50% absolute ethanol/50% hexamethyldisilazane (HMDS, Fluka Analytical, Sigma, Steinheim, Germany) and after in pure HMDS for 30 minutes each passage at RT and finally air-dried. Before observation, the samples were mounted on aluminum supports (Multilab type stub pin 1/2, Surrey, UK) using a silver paste maintaining the cell-seeding surface, coated with gold in a sputtering device (Quorum Q150RS, Technologies Ltd., Laughton, UK), and observed at 5–10 kV with a Quanta 250 (FEI Company, Milan, Italy) scanning electron microscope.

### 2.9. *In Vitro* Tube Formation Assay

Angiogenic potential to form capillary-like tubes was measured using a semisolid matrix after culturing confluent hVW-MSCs for 7 days in DMEM containing 2% FBS with 10 ng/mL HGF/SF or 50 ng/mL VEGF (Sigma); control cells were maintained in basal medium plus 10% FBS. At the end of treatments, a 96-well culture plate was coated with 50 *μ*L of Matrigel (BD Bioscence) solution for 1 hr at 37°C. After that, untreated and treated cell suspensions were placed onto the solidified layer of Matrigel at the density of 15 × 10^3^ hVW-MSCs for well. Human Umbilical Vein Endothelial Cells (HUVEC) were used as a positive control. Tubular blood vessels-like structures were observed under inverted LM and documented with a digital camera (Nikon) after 2, 6, and 24 hrs. To quantify* in vitro* angiogenesis, the number of capillary-like structures was manually counted on digitalized images taken at 4x magnification for each experimental condition. In parallel experiments, flow cytometry was performed to detect the expression of vWF, KDR, and CD31 mature endothelial cell markers in control as well as growth factors-treated cells. For surface antigen, the treated cells were rinsed in PBS, labeled with primary antibodies against KDR-APC and CD31-PE. To reveal vWF expression, the cells were fixed and permeabilized with the IntraPep Kit (Beckman-Coulter), incubated with von Willebrand Factor (vWF; Santa Cruz Biotechnology, Santa Cruz, CA, USA), and subsequently incubated with anti-mouse IgG-FITC (Dako) secondary antibody. The endothelial lineage commitment was quantified in all experimental conditions attributing a score from 0 to 4 according to the flow cytometry values of each mature endothelial cell marker. Final values are reported as mean.

### 2.10. Statistical Analysis

Results were expressed as the means ± SEM. GraphPad Prism 5.0 software (GraphPad Prism software, San Diego, CA) was used to perform statistical analysis and to create graphical representations. Statistical differences between samples were determinated using unpaired Student's* t*-test and one-way ANOVA test for comparison between more than two groups, followed by Bonferroni posttest; *p* value < 0.05 was considered to be statistically significant.

## 3. Results

### 3.1. hVW-MSC Isolation and Stemness Property

**Figure 1:**
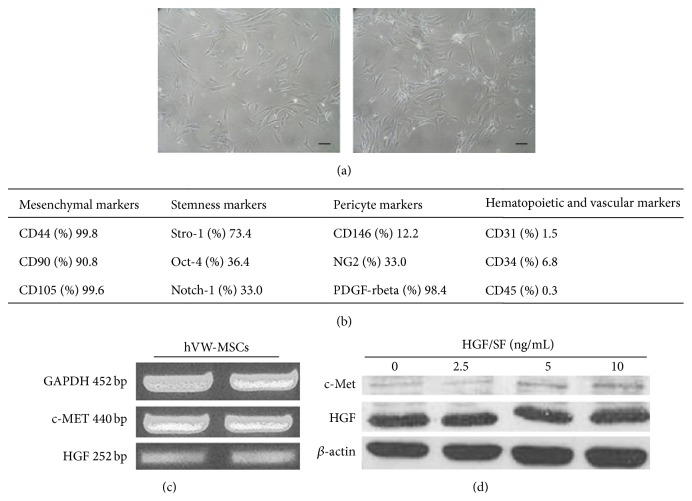
hVW-MSC aspect and HGF/*c-MET* expression. (a) hVW-MSCs were enzymatically isolated from human arteries and, at passage 3, they exhibited adherence to the plastic substrate and a typical spindle-shaped morphology. Scale bars = 100 *μ*m. (b) Flow cytometry analysis of mesenchymal, stemness, pericyte, and hematopoietic and vascular markers expressed in our cell model of hVW-MSCs. Values are reported in percentage of positivity. (c) Basal expression of HGF (252 bp) and c-MET (440 bp) mRNA in hVW-MSCs, detected by RT-PCR. (d) c-Met and HGF protein detection on cell lysates of hVW-MSCs exposed to HGF/SF (0, 2.5, 5, and 10 ng/mL) for 24 hrs.

hVW-MSCs isolated from human arteries showed a spindle-shaped morphology and a marked adhesion growth (Figure 1(a)). hVW-MSCs expressed mesenchymal (CD44, CD73, CD90, CD105, HLA-G), stemness (Stro-1, Oct-4, and Notch-1), pericyte (CD146, PDGFR-*β*, and NG2), and neuronal (nestin) markers (Figure 1(b)), together with the plasticity to differentiate in multiple mesengenic lineages, clonogenicity, and immunomodulatory functions; details on their morphology, immunophenotype, and molecular and functional features are reported elsewhere [24].

### 3.2. HGF and* c-MET* mRNA and Protein Expression in hVW-MSCs

In a previous study, Neuss et al. demonstrated that hMSCs derived from bone marrow expressed HGF and its receptor, c-Met [21]. In this study we verified whether hVW-MSCs, recovered from human arteries, also may constitutively possess these genes. Gene expression analysis showed that hVW-MSCs express HGF and* c-MET* receptor mRNA (Figure 1(c)). Western Blot confirmed the expression of the c-Met receptor in hVW-MSCs and showed increased protein levels in hVW-MSCs exposed to HGF/SF, especially at 10 ng/mL (24 hrs). Meanwhile, the expression of the HGF protein did not show evident differences under HGF/SF stimulation (Figure 1(d)).

### 3.3. HGF/SF Effect on hVW-MSC Proliferation

**Figure 2:**
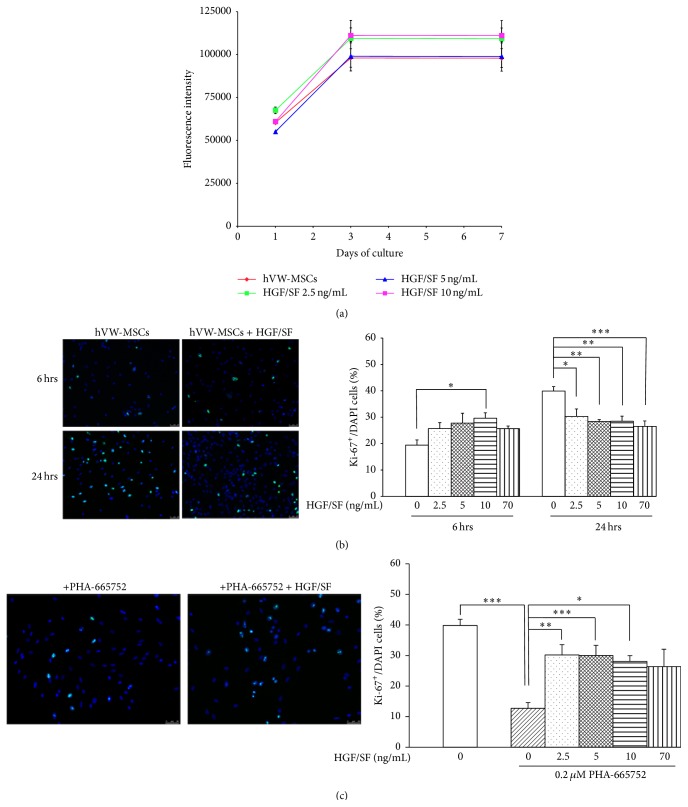
Proliferative effect of HGF/SF on hVW-MSCs. (a)* In vitro* proliferation of hVW-MSCs exposed to HGF/SF assessed by Alamar Blue fluorescence assay up to 7 days. From day 1 to day 3, the fluorescence intensity was increased in HGF/SF-treated hVW-MSCs including untreated cells (red) while no difference was seen between hVW-MSCs stimulated by HGF/SF and untreated cells at 3 and 7 days. (b) Representative images of cycling cells expressing Ki-67 protein after 6 and 24 hrs of incubation with HGF/SF, compared to controls (hVW-MSCs). Ki-67 positive cells (green) and DAPI (nuclei in blue). After 6 hrs, the HGF/SF stimulation induced a slight increase of Ki-67 positive hVW-MSCs percentage on total DAPI cells; after 24 hrs of HGF/SF exposure, the percentage of Ki-67 positive cells is approximately reduced of the 15% in all the experimental conditions. Scale bars = 50 *μ*m. (c) Images and quantitative analysis of hVW-MSCs cultured with PHA-665752 and then exposed to HGF/SF. c-Met inhibitor before HGF/SF incubation revealed almost similar Ki-67 percentage reduced in comparison to control cells. Scale bars = 50 *μ*m. (b)-(c) ^*∗*^, ^*∗∗*^, ^*∗∗∗*^ *p* value < 0.05, and one-way ANOVA test followed by Bonferroni posttest.

hVW-MSCs were exposed to 2.5, 5, and 10 ng/mL of HGF/SF for a wide range of time (from 6 hrs to 7 days). From day 1 to day 3, Alamar Blue fluorescence assay revealed an increased cell viability in all experimental conditions including control that was related to overall cell population doubling; no difference between untreated and HGF/SF-treated hVW-MSCs was seen at day 3 and day 7 (Figure 2(a)). The cell proliferation following 6 hrs and 24 hrs of exposure to 2.5, 5, 10, and 70 ng/mL HGF/SF was assayed by Ki-67 staining. Immunostaining analysis on hVW-MSCs exposed to HGF/SF for 6 hrs showed an increased Ki-67 expression in a dose-dependent manner, in comparison to the untreated hVW-MSCs; the percentage of Ki-67 positive cells was significantly higher when hVW-MSCs were exposed to HGF/SF at 10 ng/mL (29.6 ± 4.1 in HGF/SF-treated hVW-MSCs versus 19.4 ± 4.8 in untreated controls, *p* < 0.05, one-way ANOVA test followed by Bonferroni posttest). Conversely, after 24 hrs of incubation, the percentage of cycling cells positive to Ki-67 was significantly decreased in all the concentrations tested (30.3 ± 5.6 in 2.5 ng/mL, 28.4 ± 1.5 in 5 ng/mL, 28.4 ± 4.3 in 10 ng/mL, and 26.5 ± 4.5 in 70 ng/mL, versus 39.9 ± 4.0 in control cells; *p* < 0.05; one-way ANOVA test followed by Bonferroni posttest) (Figure 2(b)). hVW-MSCs exposed to PHA-665752 inhibitor showed a significant decrease of Ki-67 expression levels (12.7 ± 5.9 versus 39.9 ± 4.0 unexposed hVW-MSCs, *p* < 0.05, one-way ANOVA test followed by Bonferroni posttest). The HGF/SF addition to PHA-665752-pretreated hVW-MSCs restored the hVW-MSCs proliferation and the percentage of Ki-67 positive cells revealed overlapping results with those obtained without PHA-665752 (Figure 2(c)). Results with c-Met inhibition definitively confirmed that HGF/SF does not increase the hVW-MSCs proliferation.

### 3.4. HGF/SF Effect on hVW-MSC Migration

**Figure 3:**
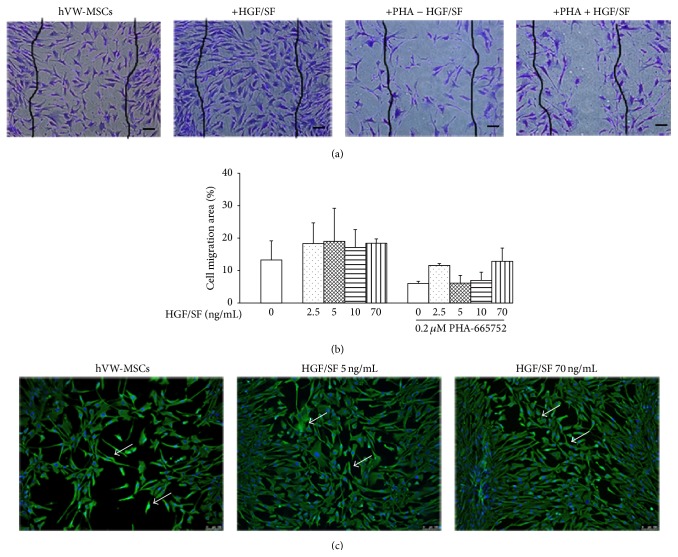
Migratory effect of HGF/SF on hVW-MSC. (a) Crystal Violet staining of hVW-MSCs migrated into the scratch area after 24 hrs of exposure to HGF/SF in comparison to the untreated hVW-MSCs; the cell migration was slackened by PHA-665752 before treatment. Wounded area is delimited by black lines. Scale bars = 50 *μ*m. (b) Quantitative analysis of cell migration area in hVW-MSCs treated with HGF/SF, in presence and absence of PHA-665752. (c) Immunofluorescence staining for Vimentin revealed that the intermediate filaments Vimentin (positive (green)) was markedly stained in migrated cells (arrows) then unmigrated cells. Scale bars = 100 *μ*m. The reported images are representative of three independent experiments.

To investigate the* in vitro* HGF/SF effect on cell migration, we used a wounded confluent hVW-MSC monolayer model cultured with or without HGF/SF for 24 hrs. hVW-MSCs exposed to HGF/SF (2.5, 5, 10, and 70 ng/mL) covered the wounded area more efficiently than untreated controls. The cell migration was reduced by adding 0.2 *μ*M of the c-Met inhibitor, PHA-665752, in the culture medium before any HGF/SF treatment. Moreover, in absence of HGF/SF stimuli, hVW-MSCs exhibited a spontaneous capacity to move into the cell-free wounded area; the spontaneous migration was probably stimulated by an autocrine mechanism related to endogenous HGF release. The addition of HGF/SF to PHA-pretreated hVW-MSCs did not completely restore the cell migration (Figures 3(a) and 3(b)). To corroborate light microscopy results, SEM investigation was performed at the same doses and time of HGF/SF treatment. SEM confirmed the high efficacy of HGF/SF to promote hVW-MSC migration into scratched area restoring the cell monolayer scratched by p200 pipette tip when compared to control cells (data not show); similar results were seen using single immunofluorescence staining for Vimentin. Notably, while Vimentin was expressed in all hVW-MSCs, the labeling fluorescence intensity was more intense in the cells migrated in the wound area; this fluorescence pattern was interpreted as a feature of cytoskeleton remodeling (Figure 3(c)).

### 3.5. HGF/SF Effect on hVW-MSC Motility

**Figure 4:**
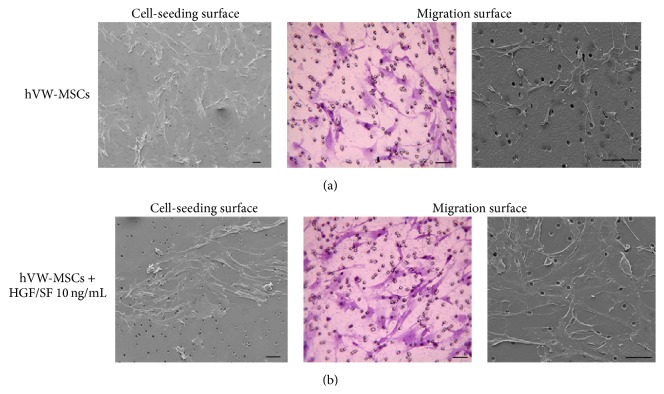
Motility effect of HGF/SF on hVW-MSCs. Representative Crystal Violet and SEM images of (a) untreated (hVW-MSCs) and (b) HGF/SF- (10 ng/mL) treated hVW-MSCs migrated through an 8 *μ*m porous membrane adhering to the migration surface. The cell-seeding surface analysis showed that the chemotactic HGF/SF facilitated the migration of many hVW-MSCs. Histological and SEM images: scale bars = 50 *μ*m.

The HGF/SF ability to mobilize and chemoattract hVW-MSCs was tested through a transwell migration assay. Crystal Violet dye showed that HGF/SF (10 ng/mL) enhanced hWV-MSC motility when compared to spontaneous hVW-MSC migration. At SEM, the majority of hVW-MSCs remained on the seeding surface in absence of HGF/SF; on the contrary, hVW-MSCs efficiently colonized the migration surface after adding 10 ng/mL of HGF/SF to the lower compartment (Figures 4(a) and 4(b)).

### 3.6. HGF/SF Effect on hVW-MSC Angiogenic Potential

**Figure 5:**
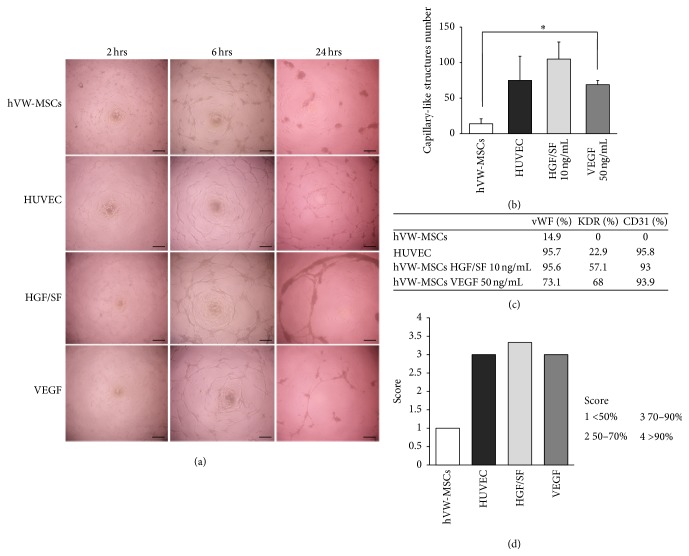
Angiogenic effect of HGF/SF on hVW-MSCs. (a) Representative* in vitro* tube-like formation images of HGF/SF (10 ng/mL for 7 days) and VEGF (50 ng/mL for 7 days) preconditioned hWV-MSCs as well as uninduced hWV-MSCs and HUVEC in Matrigel assay for 2, 6, and 24 hrs from seeding. Scale bars = 100 *μ*m. (b) The quantitative analysis of the capillary-like structures revealed a higher number of hVW-MSCs exposed to HGF/SF than VEGF-treated cells. ^*∗*^ *p* value < 0.05; Student's* t*-test. (c) Flow cytometry analysis of von Willebrand factor (vWF), KDR, and CD31 expression on untreated hVW-MSCs, HUVEC, and HGF/SF- and VEGF-treated cells. (d) The angiogenic lineage commitment was evaluated for each treatment attributing a score from 0 to 4 according to flow cytometry values.

The hVW-MSC angiogenic differentiation under the presence of HGF/SF was assessed in a 3D semisolid matrix assay. HGF/SF (10 ng/mL) or VEGF (50 ng/mL) induced the formation of capillary-like structures that peaked at 6 hrs and persisted until 24 hrs. After 2 hrs of exposure to HGF/SF or VEGF, hVW-MSCs aligned and branched from the cell periphery to form tube-like structures. At 6 hrs, the density of the HGF/SF or VEGF-induced capillary-like network becomes more evident. At 24 hrs, the number of these structures significantly decreased, except in samples treated with HGF/SF. Untreated hVW-MSCs formed very few capillary-like structures that were completely lost after 24 hrs. Human Umbilical Vein Endothelial Cells (HUVEC) were used as positive control and spontaneously formed an extensive vascular network persisting until 24 hrs (Figure 5(a)). The number of the capillary-like structures was enhanced in all experimental conditions in comparison to untreated hVW-MSCs. Although this increase was statistically significant in VEGF condition (69 ± 8.4 in VEGF 50 ng/mL versus 14 ± 9.89 in untreated controls, *p* < 0.05; Student's* t*-test), it was more pronounced after HGF/SF stimulation (Figure 5(b)). As revealed by flow cytometry analysis, the expression of mature endothelial cell markers, such as vWF, KDR, and CD31, was clearly promoted by HGF/SF or VEGF stimulation. In particular, HGF/SF exerted a greater effect on vWF expression (20% higher compared to VEGF), while KDR and CD31 were almost similar in the two treatment conditions (Figure 5(c)). The flow cytometer scores for each mature endothelial cell marker have been reported in Figure 5(d). According to the scores, hVW-MSCs preconditioned with HGF/SF and VEGF underwent a high endothelial lineage commitment that was comparable to HUVEC cells. Conversely, uninduced cells kept their mesenchymal identity.

## 4. Discussion

Ischemic foot ulcers represent a serious complication of peripheral arterial disease and the surgical revascularization remains the gold standard treatment choice, even if only 60% of cases reach an effective healing after one-year treatment [2, 25]. Wound healing is an active and complex physiological process characterized by a cascade of events such as homeostasis, inflammation, proliferation, epithelialization, and remodeling; the interplay among many cell types, growth factors, and cytokines regulates each wound healing steps to achieve a rapidly and complete wound closure [16]; any alteration of these phases may cause an ineffective tissue repair leading to chronic wounds and their management requires a very long healing time.

Many strategies have been proposed to accelerate the wound healing process; among those, proangiogenic gene and progenitor cell therapy [3, 26], engineered dermis substitute implantation [2, 4], stem cells transplantation [9–11, 27], topical application of human platelet-derived products [13–15], and growth factors such as PDGF, VEGF, TGB-beta, FGF, EGF, and GM-CFS [16, 17] have been developed.

In particular, HGF appears as an interesting multifaceted protein involved in the entire spectrum of the wound healing process [28]; HGF has been reported to participate to cell morphogenesis, motogenesis, mitogenesis, and tissue homeostasis, repair, and regeneration through the activation of its receptor, c-Met. Moreover, HGF is also endowed with antiapoptotic, anti-inflammatory, and antifibrotic activities [29] as well as proangiogenesis functions [30, 31].

In our study, we explored the HGF/SF effects on human stem cells to define a possible mechanism able to improve the wound healing process. In particular, we chose to investigate whether HGF/SF could positively influence the multipotent mesenchymal stem cells that reside within the vascular wall [24] and that have the potentiality to be mobilized in the injured tissue to contribute to better and faster wound healing.

HGF and its c-Met receptor are normally expressed by epithelial and endothelial cells [32, 33] and bone marrow mesenchymal stem cells [21–23]; here, we demonstrate that HGF and its receptor are also expressed in our cell model both at the mRNA and protein level. In particular, when hVW-MSCs cultures were exposed to HGF/SF for 24 hrs, an increase of the c-Met receptor protein levels could be detected, especially at 10 ng/mL.

The HGF effects on cell proliferation are rather heterogeneous and seem to be cell and tissue dependent [21, 30, 34–36]; few studies reported that HGF/SF inhibits mesenchymal stem cells proliferation through the cell cycle arrest [22, 37]. In this study, we observed that the administration of exogenous HGF/SF reduces hVW-MSC proliferation; the cell proliferation was assayed by using different techniques, that is, Alamar Blue and immunofluorescence. The proliferative response was not influenced by long (3 to 7 days) HGF/SF exposure time, whereas it was significantly increased by short time (6 hrs) at 10 ng/mL of HGF/SF. At 24 hrs of exposure time, hVW-MSC proliferation was significantly decreased using different concentration. Even though a slight reduction of Ki-67 nuclear expression was seen at 24 hrs of HGF/SF treatments, parallel experiments performed with the c-Met antagonist PHA demonstrated that such effect was independent of the activation of the HGF/*c-MET* axis. Substantially, our results demonstrate that HGF/SF did not exert significant effects on hVW-MSC proliferation via c-Met receptor.

The migration and the repopulation of the damaged area is a necessary step to reach the complete healing of wounds. Among the multiple biological effects of HGF, the motogenic activity appears to be one of the most powerful activities as shown in a wide range of several cell types [21, 22, 30, 34].

The scratch assay is standardized, simple, and inexpensive approach used to detect this property [38]. Accordingly, we analyzed the HGF/SF effect on wounded confluent monolayers of hVW-MSCs, by using several morphological approaches including histological staining, SEM,and immunofluorescence. Our results demonstrated that different doses of HGF/SF augmented the hVW-MSC ability to migrate in the cell-free area restoring the wounded cell monolayer. Moreover, these migrating cells expressed more intensely Vimentin, an intermediate filaments marker; this feature could be related to the cytoskeleton remodeling phenomenon. The hVW-MSC migration was reduced in the presence of the c-Met receptor inhibitor, PHA-665752, even if the HGF/SF addition did not induce the complete repopulation of the wounded area. As regard the untreated hVW-MSCs, we saw spontaneous migration, contrary to literature, probably related to the autocrine secretion of endogenous HGF released by the same multipotent cells.

The chemoattractive property of HGF/SF was confirmed through the transwell migration assay. The histological and ultrastructural observations demonstrated that hVW-MSCs were able to cross the porous membrane, as attracted by HGF/SF placed at underlying bottom, and colonize the layer directly in contact with the growth factor. In the clinical context, it could be therefore speculated that a steady disposal of HGF/SF in the wound could recruit endogenous stem cells, thus promoting tissue repair.

Therapeutic angiogenesis represents another crucial event in wound healing process. VEGF is a well-established proangiogenic factor in promoting blood neovessels formation and angiogenesis [39]. In our previous studies [24, 40], VEGF has been demonstrated to be highly effective in promoting hVW-MSC endothelial cell lineage commitment. In that regard, also HGF was proposed as a powerful angiogenic promoter [30, 31, 41] able to stimulate endothelial cell proliferation, alignment, and organization into blood vessel-like structures, although it was originally discovered as a potent growth factor for liver regeneration [29]. We assessed the HGF/SF angiogenic potential on our cell model through an* in vitro* 3D angiogenesis assay using Matrigel; we succeeded in demonstrating that HGF/SF pretreated hVW-MSCs possess a high ability to form an extensive capillary-like network. Remarkably, the HGF/SF angiogenic effect on hVW-MSCs was comparable to that seen in the positive control (HUVEC) and with the VEGF pretreatment; following HGF/SF (10 ng/mL) stimulation, the resulting capillary-like structures persisted even at 24 hrs, suggesting that the HGF/SF could act on the vascular integrins or adhesion molecules, therefore stabilizing the endothelial cell junctions. HGF/SF and VEGF growth factors greatly increased the expression of vWF, KDR, and CD31, typical mature endothelium markers, supporting an endothelial cell lineage commitment.

According to our data, the population of vascular mesenchymal stem cells basically expresses the HGF and its receptor, c-Met. Interestingly, the HGF/SF addition was shown to enhance the hVW-MSC migration and motility as well as the capillary-like structures formation. These data suggest that a novel therapeutic strategy, based on the local delivery of HGF/SF and the involvement of the vascular wall-MSCs, could be developed to accelerate the healing of unresponsive vascular ulcers.

## Figures and Tables

**Table 1:** List of primers used for RT-PCR in hVW-MSCs.

Gene	Primer sequence	Product size (bp)	*T* (°C)
GAPDH	FWD 5′-ACCACAGTCCATGCCATCAC-3′	452	61
	REV 5′-TCCACCACCCTGTTGCTGTA-3′		

c-MET	FWD 5′-AGAAATTCATCAGGCTGTGAAGCGCG-3′	440	68
	REV 5′-TTCCTCCGATCGCACACATTTGTCG-3′		

HGF	FWD 5′-TTTGCCTTCGAGCTATCGGG-3′	254	62
	REV 5′-GCAAGAATTTGTGCCGGTGT-3′		
